# Metabolic shift during fermentation in kimchi according to capsaicinoid concentration

**DOI:** 10.1016/j.heliyon.2024.e24441

**Published:** 2024-01-11

**Authors:** Young Bae Chung, Sung Jin Park, Yun-Jeong Choi, Ye-Rang Yun, Mi-Ai Lee, Sung Hee Park, Sung Gi Min, Hye-Young Seo

**Affiliations:** Research and Development Division Kimchi, World Institute of Kimchi, Gwangju, 91755, Republic of Korea

**Keywords:** Red-pepper, Capsaicinoids, Kimchi, Fermentation, Metabolites

## Abstract

The cultivar of red pepper used in kimchi contributes to spiciness, red color, and fermentation characteristics. Capsaicinoids are the main components of red pepper. Therefore, understanding changes in metabolites during kimchi fermentation according to capsaicinoid concentration is necessary to control the quality of kimchi. The purpose of this study was to investigate the effect of capsaicinoids on metabolites during kimchi fermentation. To profile the effect of capsaicinoid concentrations on kimchi fermentation, five kimchi samples were prepared using different concentrations of capsaicinoids (4, 12, 30.7, 40.9, and 50.3 mg/kg) and stored at 4 °C for 28 days. During kimchi fermentation, pH, titratable acidity, capsaicinoid concentration, total viable and lactic acid bacteria, free sugars, amino acids, and microbial community were evaluated. Each result was statistically analyzed for changes in capsaicin concentration and fermentation time. The capsaicinoid concentration did not change during kimchi fermentation but the growth of lactic acid bacteria changed. According to the growth of lactic acid bacteria, free sugar, amino acids, and microbial community changed with the capsaicinoid concentration. Overall, the results of this study provide preliminary information on the use of red pepper and capsaicinoids in the kimchi industry.

## Highlights

• Capsaicinoids alter lactic acid bacteria abundance and diversity in kimchi.
• Capsaicinoids lower pH and raise titratable acidity in kimchi samples.
• Capsaicinoids increase organic acid and free amino acid contents in kimchi samples.
• Capsaicinoid addition alters bacterial succession in kimchi samples.

## 1 Introduction

Kimchi is a representative fermented food and has been consumed for thousands of years in Korea [1]. For storage without spoilage, seasonal vegetables are salted, mixed with other vegetables, and stored in airtight containers at low temperatures [2]. These processes during kimchi preparation spontaneously generate lactic acid bacteria, imparting a unique taste and health benefits [3]. The quality of kimchi depends on the freshness of raw ingredients, optimal processing, and fermentation conditions [4]. Among the various factors that characterize kimchi, red pepper is unique as it affects the spicy taste, color, flavor, and microorganism growth, thus determining the quality of kimchi [5,6]. Capsaicinoids, synthesized in the placenta and septum of red pepper, are key bioactive compounds that impart pungency and exert antimicrobial effects [7]. In red pepper, capsaicin and dihydrocapsaicin are two major capsaicinoids, accounting for 77–98 % of their pungency [8]. Several studies have demonstrated the effects of red pepper content on spiciness and alteration in the abundance of lactic acid bacteria (LAB) in kimchi along with the correlation between capsaicinoids concentration and physicochemical properties of kimchi, such as spiciness, and changes in the composition of microbes and free sugar [9,10]. However, as the metabolites changes during kimchi fermentation are diverse, more evidence from metabolite analysis is needed to evaluate the effect of capsaicinoids. In this study, we investigated the effect of capsaicinoids on the primary and secondary metabolites during kimchi fermentation. Additionally, the concentration of capsaicinoids in kimchi fermentation and its impact on quality were evaluated by fixing the amount of red pepper powder and fermentation conditions while adjusting the capsaicin concentration by varying red pepper varieties.

## 2 Materials and methods

### 2.1 Materials

Salted kimchi cabbage, radish, green onion, garlic, ginger, anchovy sauce, glutinous rice, vegetable broth, sugar, salt, and red pepper powder were purchased from a local market in Gwangju, Republic of Korea.

### 2.2 Kimchi preparation

Four types of red pepper powder were mixed and used according to the difference in the concentration of capsaicinoids. Briefly, salted kimchi cabbage (8.82 kg) was cut into 2.5 cm × 2.5 cm pieces and mixed with radish (1.11 kg), green onion (0.40 kg), garlic (0.27 kg), ginger (0.07 kg), anchovy sauce (0.21 kg), glutinous rice (0.43 kg), vegetable broth (0.37 kg), sugar (0.11 kg), salt, (0.03 kg), red-pepper powder (0.6 kg), and water (0.24 kg). The final concentrations of capsaicinoids in kimchi samples were 4, 12, 30.7, 40.9, and 50.3 mg/kg. The kimchi samples were packed in a polyethylene bag and fermented at 4 °C for 28 days. The independent variable in this study was the capsaicin content depending on the red pepper variety, and the dependent parameters included raw materials other than red pepper powder and fermentation conditions.

### 2.3 pH and titratable acidity

Kimchi (100 g) was homogenized using a hand blender (No. MQ775; Braun Korea, Seoul, Korea). The pH of the filtrate was measured using a pH meter (Thermo Fisher Scientific Beverly, MA, USA). Titratable acidity was measured by titrating 10 mL kimchi juice with 0.1 N NaOH to pH 8.3, concerting the volume of alkali solution used into the lactic acid content (%, w/w), and expressing this value as the titratable acidity (%).

### 2.4 Microbial analysis

Kimchi (10 g) was added to 100 mL of sterile saline (0.85 % w/v NaCl), and kimchi juice was prepared in a stomacher (Bagmixer R 400; Interscience, Saint-Nom-la-Bretèche, France). The kimchi juice was diluted tenfold with sterile saline, dispensed into each medium, and cultured at 37 °C for 48 h. The microbial colonies were then enumerated. Total bacterial counts and lactobacilli were enumerated using 3 M Petrifilm (3 M CO., MN, USA).

### 2.5 Capsaicinoids analysis

For the extraction of capsaicinoids, homogenized kimchi juice (1 mL) was mixed with 5 mL of acetone and a vortex mixer for 2 min. The extract was then dried under a nitrogen stream, dissolved in acetonitrile, and subjected to C18 Sep-Pak. The capsaicin and dihydrocapsaicin concentrations were measured using a high-performance liquid chromatography (HPLC) system (Agilent 1100 series; Agilent Technologies, Palo Alto, CA, USA). The separation was performed using a Lachrom Ultra C18 column (50 mm × 2.0 mm; 2 μm; Hitachi, Tokyo, Japan). The mobile phase comprised a 4:6 (v/v) mixture of acetonitrile and 0.1 % (v/v) acetic acid, the flow rate was 0.6 mL/min, and the sample injection volume was 2 μL. The capsaicinoids were quantitated in a spectrofluorometric detector at 280 nm excitation and 325 nm emission. HPLC with fluorescence detection with 90 % (v/v) HPLC with fluorescence detection and 90 % (v/v) HPLC-grade methanol was used to quantify the capsaicin, dihydrocapsaicin, and total capsaicinoid (sum of the capsaicin and dihydrocapsaicin) concentrations.

### 2.6 Free sugar analysis

Homogenized kimchi juices were centrifuged at 10,000×*g* for 15 min and the supernatants were passed through 0.45 μm membrane filters (Toyo Roshi Kaisha Ltd., Tokyo, Japan). The free sugar content in each filtrate was measured using HPLC (Agilent 1100 series; Agilent Technologies, Palo Alto, CA, USA) fitted with a carbohydrate analysis column (4.6 mm × 250 mm; Waters Co., Milford, MA, USA). The mobile phase was 80:20 (v/v) acetonitrile: water, the flow rate was 1.0 mL/min, the injection volume was 20 μL, and a refractive index detector (RID) was used (Agilent 1100 series; Agilent Technologies). Glucose, fructose, and mannitol were the standards.

### 2.7 Organic acid analysis

For the extraction of organic acid in kimchi, homogenized kimchi (2 g) was diluted with distilled water (5 mL) and the mixture was extracted using a sonicator (PowerSonic 520; Hwashin Tech Co., Daegu, Republic of Korea) for 30 min. The extracts were passed twice through filter papers (Advantec No. 1; Toyo Roshi Kaisha Ltd.) and syringe-filtered (0.2 μm × 25 mm; Sartorius, Gyeonggi, Republic of Korea). HPLC was conducted using an Agilent 1260 Infinity/G4212B system (Agilent Technologies) fitted with a variable wavelength diode array detector set to 210 nm. The injection volume was 10 μL. The organic acids were analyzed in an Aminex HPX-87H column (300 mm × 7.8 mm; 9 μm, Bio-Rad Laboratories, Hercules, CA, USA) at 50 °C. Isocratic elution was performed using a mobile phase consisting of 0.008 N H2SO4 in deionized water. The elution time was 30 min and the flow rate was 0.6 mL/min. The organic acids in the samples were identified by comparing their retention times against those of standard organic acids and quantified by interpolation of a calibration curve plotted from the peak areas for the organic acid standards.

### 2.8 Free amino acid analysis

One gram of each homogenized kimchi sample was placed in a 50 mL centrifuge tube and the volume was adjusted to 10 mL with distilled water. Each suspension was centrifuged at 3000 rpm for 30 min, 1 mL of 5 % (v/v) trichloroacetic acid (TCA) was added to 1 mL of supernatant, and the mixture was centrifuged at 6800 rpm for 20 min. The supernatant was passed through a syringe filter (0.2 μm × 25 mm; Sartorius) and evaluated in an automatic amino acid analyzer (L-8900; Hitachi). An ion exchange column (4.6 mm × 60 nm; Hitachi HPLC pack column. No. 2622 S C PF) was used for the analysis. Amino acids in the sample were quantitated quantified using a UV detector at 570 nm excitation and 440 nm emission. The mobile phase was analyzed with Wako L-8900 buffer solution (Wako Pure Chemical Industries Ltd., Osaka, Japan). The flow rate was 0.35 mL/min, and the injection volume was 20 μL.

### 2.9 Microbial diversity analysis

Microbial DNA was extracted using a DNeasy PowerSoil kit (Qiagen, Hilden, Germany) according to the manufacturer's instructions and quantified using Quant-IT PicoGreen (Invitrogen, Carlsbad, CA, USA). The sequencing libraries were prepared according to Illumina 16 S metagenomic sequencing library protocols (Illumina, San Diego, CA, USA) to amplify the bacterial V3 and V4 regions. Two nanograms of input gDNA genomic DNA was amplified using PCR in 5× reaction buffer, 1 mM dNTP mix, 500 nM of each universal F/R PCR primer, and Herculase II fusion DNA polymerase (Agilent Technologies). The first PCR cycle comprised heat activation at 95 °C for 3 min, 25 cycles at 95 °C for 30 s, 55 °C at 30 s, 72 °C at 30 s, and a final extension at 72 °C for 5 min. The universal primer pair with Illumina adapter overhang sequences used in the initial PCR was as follows: (V3–F: 5′-TCGTCGGCAGCGTCAGATGTGTATAAGAGACAGCCTACGGGNGGCWGCAG-3′ and V4-R:5′-TCGTCGGCAGCGTCAGATGTGTATAAGAGACAGCCTACGGGNGGCWGCA G-3′ and V4-R:5′-TCGTCGGCAGCGTCAGATGTGTATAAGAGACAGCCTACGGGNGG.

CWGCAG-3′). The first PCR product was purified using AMPure beads (Agencourt Bioscience, Beverly, MA, USA), and 2 μL of purified PCR product was amplified using PCR for final library construction with the index added via a Nextera XT library preparation kit (Illumina). The second PCR cycle was run under the same conditions as the first, except there were 10 cycles, and the PCR product was purified using AMPure beads. The purified PCR product was quantified using qPCR according to the qPCR quantification protocol guide and a KAPA library quantification kit for Illumina sequencing platforms. The purified PCR product was then subjected to quality control with using TapeStation D1000 screen tape (Agilent Technologies, Waldbronn, Germany). Paired-end (2 × 300 bp) sequencing was performed on the MiSeq™ platform (Illumina) by Macrogen (Seoul, Republic of Korea).

### 2.10 Statistical analysis

All experiments were performed in triplicate, and the results were expressed as means ± standard deviation (SD). One-way analysis of variance (ANOVA), *t*-test, and Duncan's multiple-range comparison tests were performed at the 0.05 % level in SPSS software (Statistical Package for Social Sciences, version 19, SPSS Inc., Chicago, IL, USA).

## 3 Results and discussion

### 3.1 Influence of capsaicinoids in kimchi on fermentation parameters

Kimchi processing does not include a sterilization; therefore, it continues to ferment owing to microorganisms originating from vegetables and raw materials during storage [11]. Fermentation alters the characteristics of kimchi, such as the pH, titratable acidity, total viable bacteria and LAB, free sugar, organic acids, abundance of various metabolites, and amino acid contents [12]. Therefore, we evaluated the effect of capsaicinoids on the fermentation characteristics of kimchi stored at 4 °C for 28 days.

**Fig. 1:**
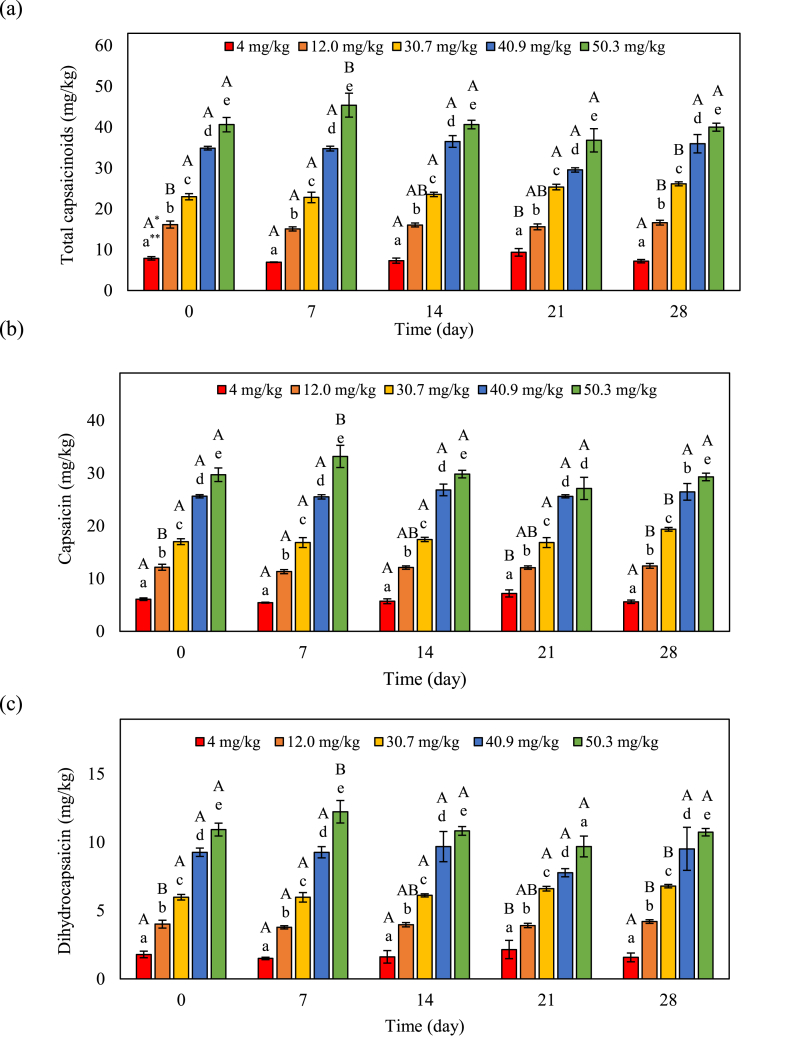
Changes in capsaicinoids during fermenting kimchi. (a) Total capsaicinoids, (b) capsaicin, and (c) dihydrocapsaicin. The error bars represent the standard deviation of the mean (n = 3). *Uppercase letters indicate a significant difference (*p* < 0.05) within the same sample over time. **Lowercase letters indicate significant differences (*p* < 0.05) among the treatments at the same time.

The total capsaicinoids, capsaicin, and dihydrocapsaicin concentrations in different kimchi samples are shown in Fig. 1a–c. Total capsaicinoids, capsaicin, and dihydrocapsaicin concentrations in kimchi samples did not change during fermentation. No concentration dependent changes were observed. These results indicate that spontaneous kimchi fermentation was not affected by capsaicinoids concentrations. Similarly, Park et al. [6] investigated five sets of kimchi samples according to capsaicinoids concentration and did not observed a change in capsaicinoid concentrations. This phenomenon may be due to the hydrophobicity of capsaicinoid compounds. Capsaicinoids are lipid-soluble alkaloids that are synthesized in the placenta and septum of red pepper [7]. Cho et al. [13], reported that the degradation of capsaicinoids of red pepper juice was observed during a fermentation period of 5 days. Therefore, the decomposition of capsaicinoids in red pepper powder is delayed by fermentation.

**Fig. 2:**
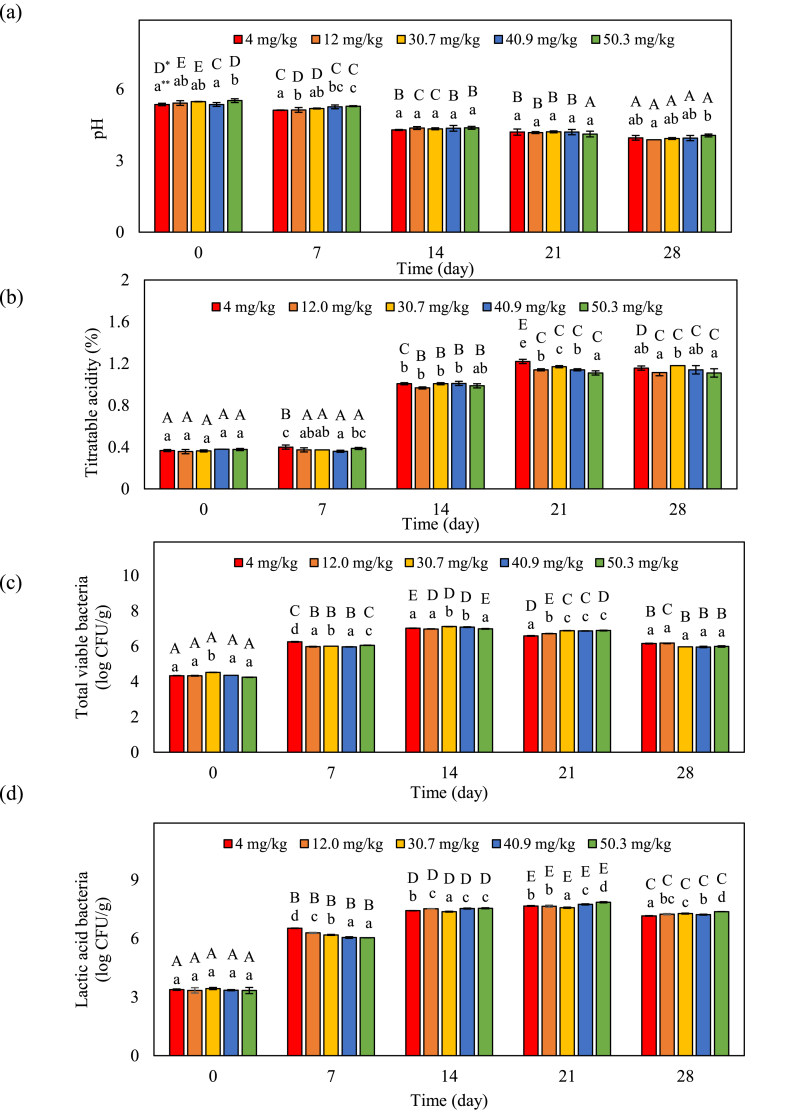
Changes in pH (a), titratable acidity (b), total viable bacteria (c), and LAB (d) with capsaicinoids levels and fermentation. The error bars represent the standard deviation of the mean (n = 3). *Uppercase letters indicate a significant difference (*p* < 0.05) within the same sample over time. **Lowercase letters indicate significant differences (*p* < 0.05) among the treatments at the same time.

Changes in pH, titratable acidity, total viable bacteria, and LAB in the kimchi samples at different time points of fermentation are shown in Fig. 2a–d. On fermentation day 0, the pH of kimchi was between 5.36 and 5.52 depending on the capsaicinoid concentration. However, there was no significant change with increasing capsaicinoids concentration after 14 days. Finally, the pH of all kimchi samples, except for those treated with 50.5 mg/kg capsaicinoids, decreased to below pH 4.0 (Fig. 2a). Chung et al. [10] reported that for different kimchi samples, the pH sharply decreased in early stages of fermentation but gradually decreased after that. Moreover, Park et al. [6] demonstrated that capsaicinoid addition significantly decreases kimchi pH. Continuously, titratable acidity of all kimchi samples rapidly increased during fermentation regardless of capsaicinoid content (Fig. 2 b). Changes in pH and titratable acidity are closely associated with the lactic acid bacteria (LAB) involved in kimchi fermentation [4]. Jeong et al. [14] reported that the changes in kimchi pH and titratable acidity increase with the storage period. Here, however, pH and titratable acidity did not significantly differ with capsaicinoid concertation among kimchi samples. This could be owing to differences in seasoning composition, the amount of red pepper powder used, and the amount of other seasoning ingredients used according to the capsaicinoid ratio.

Although change in pH and titratable acidity did not significantly differ with capsaicinoid concentration among kimchi samples, the capsaicin concentration was found to be involved in microbial changes (Fig. 2 c, d). For all kimchi samples, the initial viable bacterial counts were 4.24–4.52 log CFU/g. The total viable bacterial count rapidly increased until day 14 of fermentation and decreased thereafter. By day 14 of fermentation, the highest total aerobic bacterial counts were in the range of 6.98–7.11 log CFU/g for all kimchi samples (Fig. 2c). Changes in LAB were affected by capsaicinoids (Fig. 2d). LAB abundance in all kimchi samples rapidly increased until day 21 of fermentation and decreased. After 28 days, LAB abundance again increased with increasing capsaicinoid concentration. As fermentation continued in the presence of different capsaicinoid concentrations, LAB growth significantly changed. Previous studies have shown that capsaicinoid concentrations have an influence on LAB during kimchi fermentation [6,10,14]. Therefore, as a result of this study, the capsaicinoid content may have an effect on the growth of LAB.

### 3.2 Effect of capsaicinoids in kimchi on metabolic shift

**Fig. 3:**
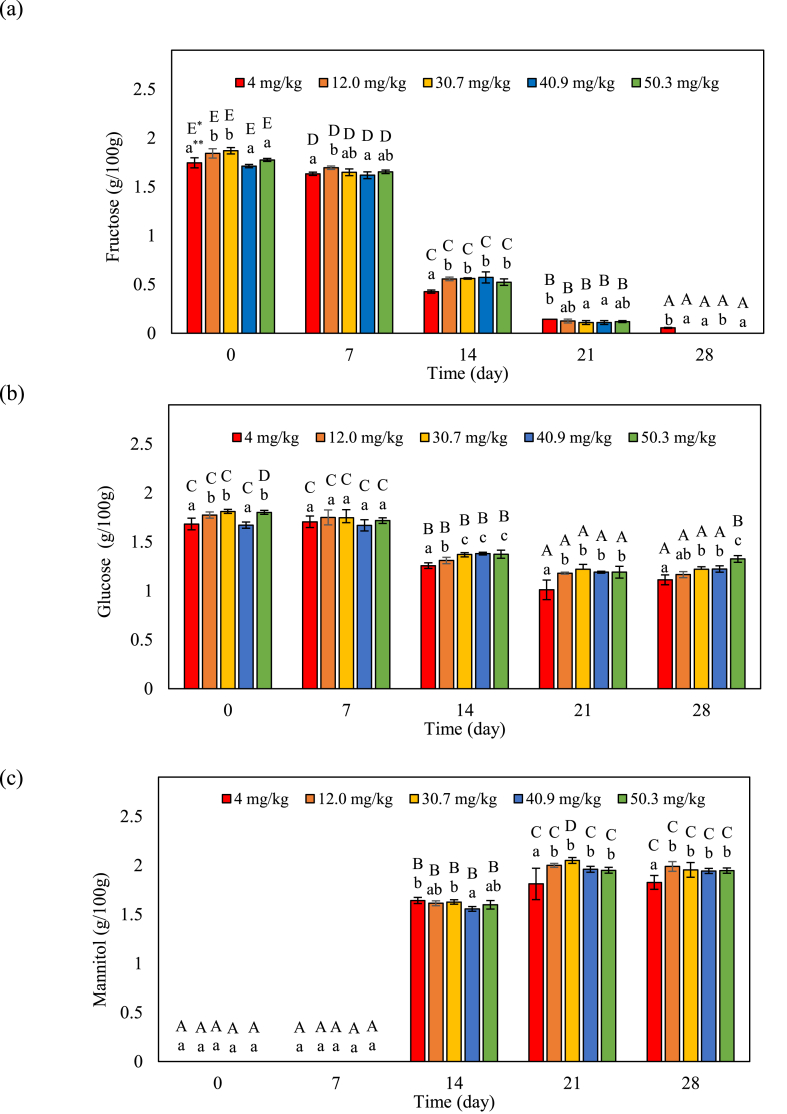
Changes in fructose (a), glucose (b), and mannitol (c) content with capsaicinoids levels and fermentation. The error bars represent the standard deviation of the mean (n = 3). *Uppercase letters indicate a significant difference (*p* < 0.05) within the same sample over time. **Lowercase letters indicate significant differences (*p* < 0.05) among the treatments at the same time.

Based on the results of 3.1 section, the contents of free sugars, organic acids, and amino acids during fermentation were investigated. Changes in the free sugar content of fermenting kimchi samples containing different concentrations of capsaicinoids are shown in Fig. 3 a-c. Free sugar, a carbon source for the bacteria that ferment kimchi, is produced by the degradation of the polysaccharide in the cell walls of kimchi cabbage [15]. At the start of fermentation, there was no change in fructose content according to capsaicin concentration. The fructose content sharply decreased in all kimchi samples until day 14 of fermentation and was undetectable thereafter in all samples except kimchi-A until the end of fermentation (Fig. 3 a). Similarly, the glucose content decreased in all kimchi samples as fermentation progressed (Fig. 3 b). In contrast, mannitol content increased after day 14 of fermentation, and more mannitol was measured in kimchi with higher capsaicinoids concentration after 28 days. A previous study on kimchi fermentation showed that LAB use fructose as a carbon source and convert. It to mannitol, which is a sugar alcohol that significantly influences kimchi flavor [16]. In heterolactic fermentation, LAB and certain other bacteria generate mannitol when utilizing fructose as an electron acceptor [10].

**Fig. 4:**
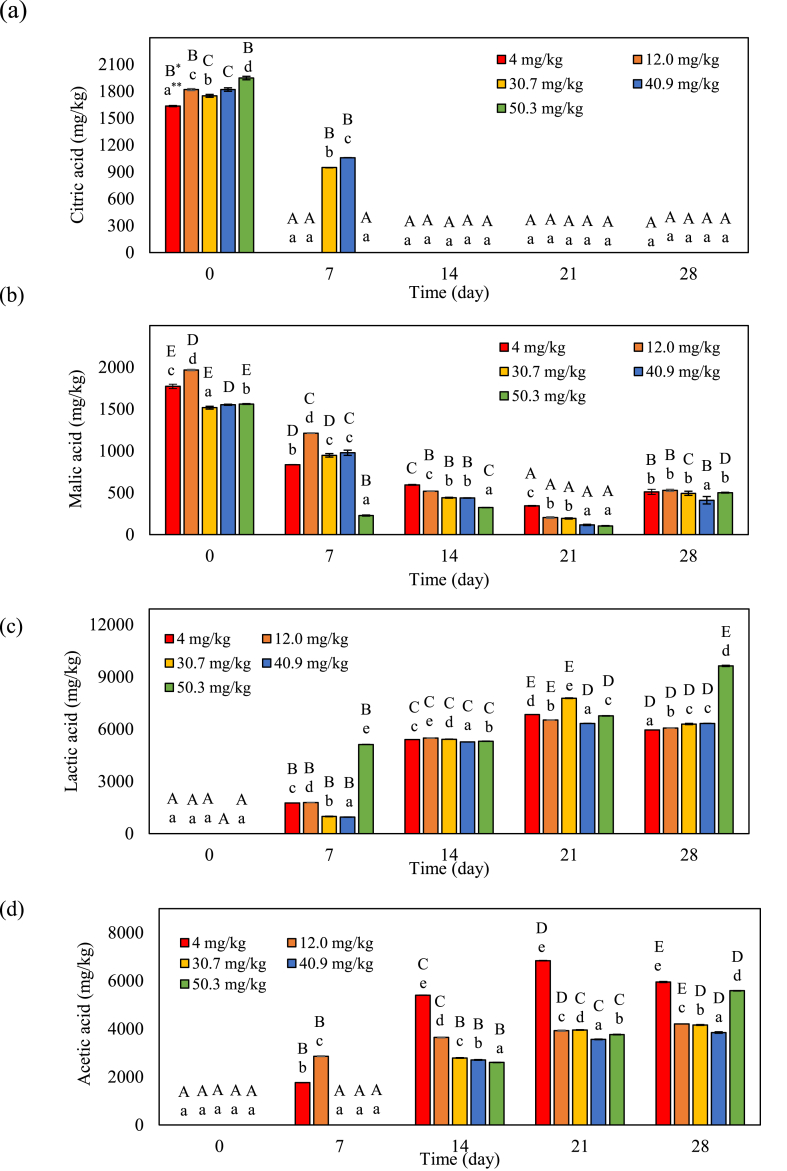
Changes in citric acid (a), malic acid (b), lactic acid (c), and acetic acid (d) in kimchi samples with different capsaicinoids levels and stages of fermentation. **Uppercase letters indicate a significant difference (*p* < 0.05) within the same sample over time. **Lowercase letters indicate significant differences (*p* < 0.05) among the treatments at the same time.

Changes in organic acids contents among kimchi samples during fermentation are shown in Fig. 4 a-d. Citric acid and malic acid decreased, whereas lactic acid and acetic acid contents increased. Citric acid in all kimchi samples was not detected after 14 days of fermentation (Fig. 4a). The malic acid content substantially decreased as fermentation progressed (Fig. 4b). Park et al. [17] and Yoo et al. [18] reported that malic acid and citric acid are converted by LAB to lactic and acetic acids during kimchi fermentation. After day 28 of fermentation, lactic acid and acetic acid content increased in all kimchi samples (Fig. 4 c, d). During fermentation, lactic acid content was highest in kimchi with high capsaicinoids (Fig. 4c), and acetic acid content was highest in kimchi with low capsaicinoid concentrations. According to Park et al. [17], the elevated lactic acid and acetic acid production observed in kimchi containing capsaicinoids may be the result of bacterial fermentation in the presence of high sugar and nutrient levels during the early fermentation stages.

**Fig. 5:**
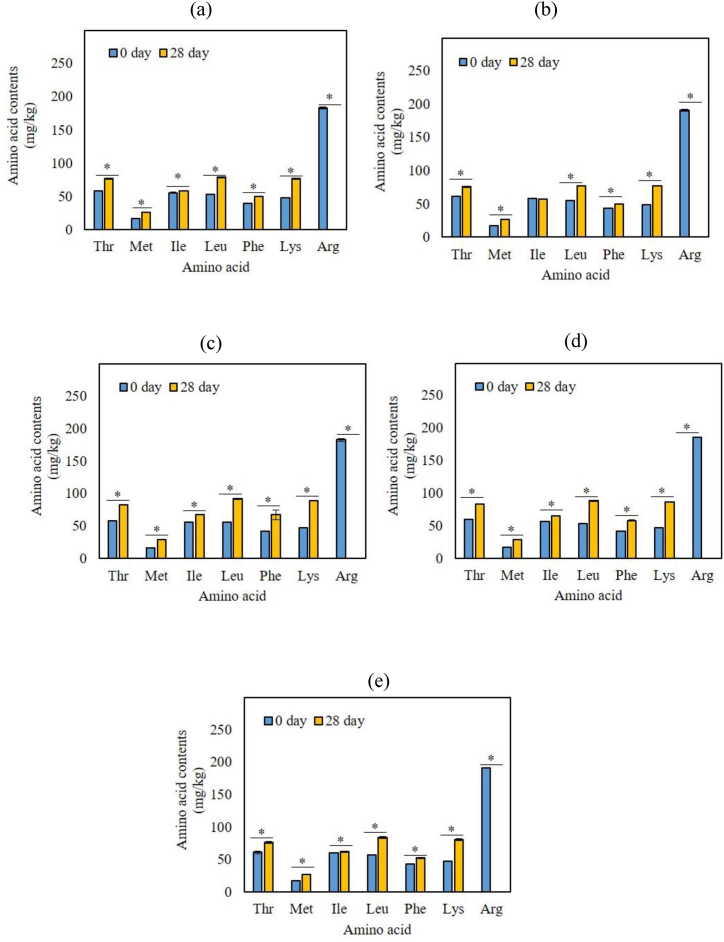
Changes in essential amino acids in kimchi containing different levels of capsaicinoids: 4 mg/kg (a), 12 mg/kg (b), 30.7 mg/kg (c), 40.9 mg/kg (d), and 50.3 mg/kg (e). Thr: Threonine; Met: Methionine; Ile: Isoleucine; Leu: Leucine; Phe: Phenylalanine; Lys: Lysine; Arg: Arginine. Data shown represent means ± SD; n = 3. *p < 0.05 within the same sample over time.

Free amino acids are also important contributors to the flavor and taste of fermented kimchi. The observed changes in amino acid content in the kimchi samples are shown in Fig. 5 and Table 1. Essential amino acids, such as threonine (Thr), methionine (Met), isoleucine (Ile), leucine (Leu), phenylalanine (Phe), lysine (Lys), and arginine (Arg) were observed in all kimchi samples (Fig. 5). Non-essential amino acids were shown in Table 1. Essential amino acids, such as threonine (Thr), methionine (Met), isoleucine (Ile), leucine (Leu), phenylalanine (Phe), lysine (Lys), and arginine (Arg), were observed in all kimchi samples (Fig. 5). Non-essential amino acids are shown in Table 1. The contents of all essential amino acids except Arg were increased (Fig. 5). The results indicated that the changes in essential amino acid contents were more affected by fermentation than by capsaicinoid concentration. The content of non-essential amino acids, such as aspartic acid, glutamic acid, glycine, alanine, valine, γ-aminobutyric acid (GABA), and ornithine increased until day 28 of fermentation (Table 1). The level of several amino acids rapidly increases early in fermentation and decreases by the fermentation midpoint [13,18]. Fish sauce mainly contains glutamic acid, valine, and arginine, which could support LAB growth during the initial fermentation stages; additionally, LAB themselves may produce the enzymes required to generate and replenish these amino acids [19,20]. Overall, the free amino acid level varies with kimchi and additive composition as well as fermentation period and temperature [21].

**Table 1:**
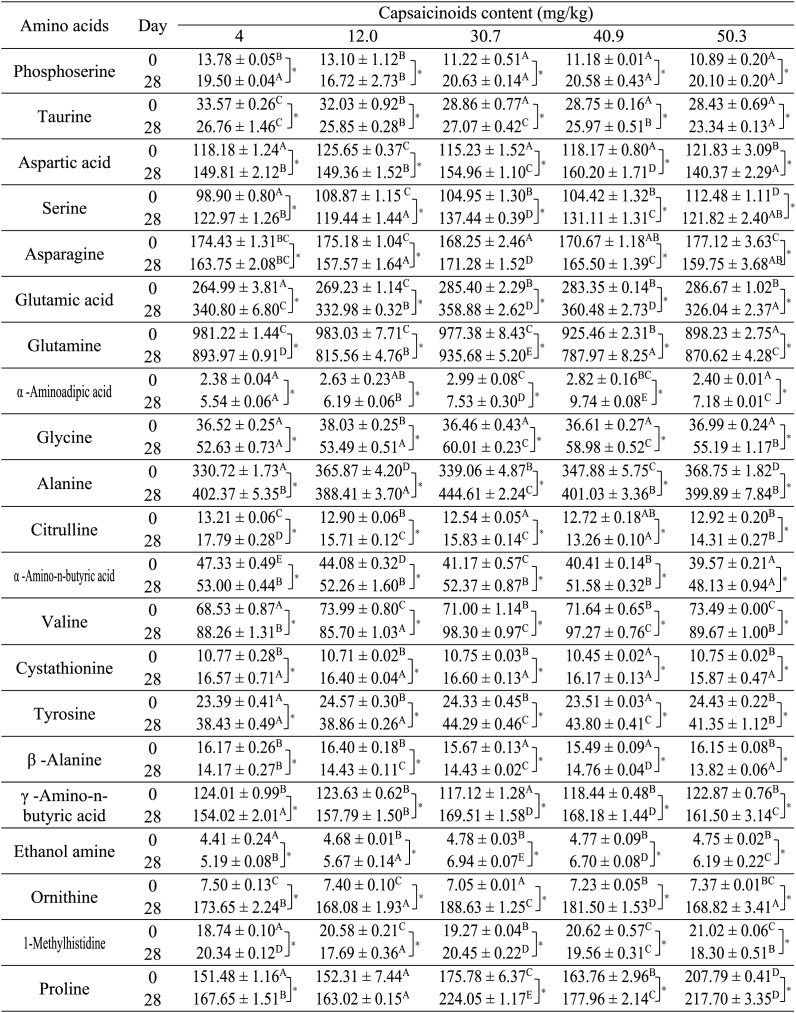
Changes in nonessential amino acids contents of kimchi samples during the fermentation period at different capsaicinoids content. Results showing one-way ANOVA (uppercase) and T-test (asterisk) analysis. The error bars represent standard deviation of mean (n = 3). Uppercase indicate significant difference (p < 0.05) among the treatments within same time point. And asterisks indicate the significant difference (p < 0.05) within same sample over time.

### 3.3 Changes in microbial diversity during kimchi fermentation

**Fig. 6:**
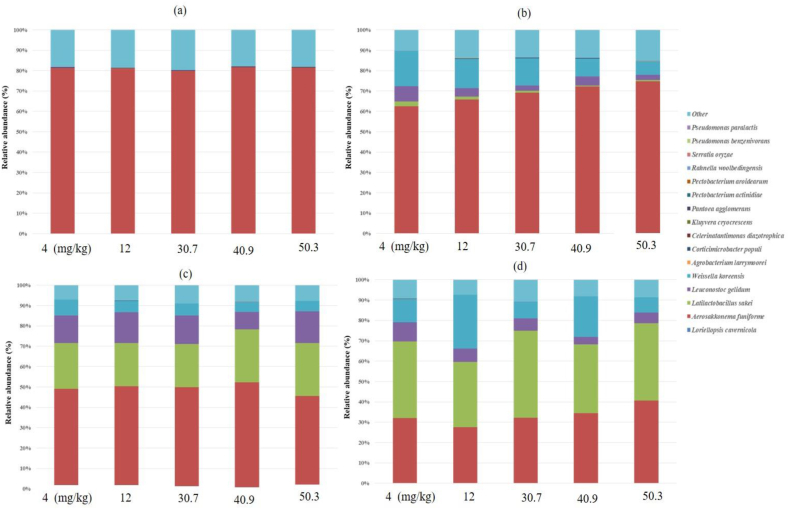
Bacterial species composition in kimchi samples on day 0 (a), day 7 (b), day 14 (c), and day 28 (d) of fermentation. “Other” includes bacterial genera compromising <1.0 % of all high-quality reads.

Bacterial species diversity was compared among fermented kimchi samples differing in capsaicinoid concentration (Fig. 6a–d). *Aerosakkonema funiforme* and *Celerinatantimonas diazotrophica* were the major strains in all kimchi samples early in fermentation (Fig. 6a). *Aerosakkonema funiforme* spontaneously grew in the early stage of fermentation but gradually decreased as fermentation progressed. It is the dominant strain in kimchi that is naturally fermented without starter [22]. The microbial diversity of fermented kimchi varies with the additives used and fermentation temperature and duration. By day 7 of fermentation, *Weissella koreensis* was detected in all kimchi samples and the proportion of W. *Koreensis* was highest in kimchi containing 4 mg/kg capsaicinoids (Fig. 6b). Similarly, a previous study confirmed that capsaicinoid addition strongly influences *W. koreensis* abundance during kimchi fermentation regardless of fermentation temperature or duration. *W. koreensis* populations rapidly increased early in fermentation and remained constant thereafter, regardless of temperature [23]. Here, the original bacterial community in the early stages of kimchi fermentation was rapidly replaced by LAB, including *Latilactobacillus sakei*, *Leuconostoc gelidum*, and *W. koreensis* at the fermentation midpoint (Fig. 6c). Similar findings were reported previously [13,22]. As fermentation progressed, acid-tolerant LAB such as *L. sakei*, *W. koreensis*, and *L. gelidum* rapidly predominated, and their abundances were relatively stable until the late stage of kimchi fermentation. The abundance of *L. sakei* rapidly increased in all kimchi samples after 28 days (Fig. 6d). A previous study reported that the relative abundance of *L. gelidum* decreases at the fermentation midpoint, whereas that of *L. sakei* continues to rise rapidly even during late fermentation [24]. The rapid growth of LAB and extreme conditions of kimchi fermentation may have inhibited the growth of *Aerosakkonema funiforme*. Heterofermentative LAB such as *Leuconostoc* spp. predominate under the mildly acidic and less anaerobic conditions of early kimchi fermentation. In contrast, homofermentative LAB such as *Latilactobacillus* spp. and *Weissella* spp. predominate later in kimchi fermentation when the conditions are more anaerobic and acidic [25,26]. Hence, the microbial compositions were probably similar for all kimchi samples. However, the microbial dynamics were slightly different among samples during kimchi fermentation.

## 4 Conclusion

In summary, the present study evaluated the effects of capsaicinoids on the metabolite profiles and microbial community of fermenting kimchi. The findings demonstrated changes in free sugar, organic acid, and free amino acid levels among fermented kimchi samples. Free amino acids, such as GABA and ornithine, were produced during kimchi fermentation. However, the metabolite levels did not significantly differ among kimchi samples with varying capsaicinoid concentrations. Changes in the metabolite profile of kimchi were dependent on the capsaicinoid concentration and fermentation. Moreover, the composition of microbial communities was similar across kimchi samples. However, the microbial dynamics slightly differed among samples as fermentation progressed. The addition of red pepper powder with different capsaicinoid concentrations to kimchi altered microbial dynamics and metabolite profiles at different stages of fermentation and, therefore, affected the taste and flavor of the final fermented kimchi product.

## Funding statement

This research was supported by the World Institute of Kimchi (KE2102-2-2 & KE2402-1), funded by the Ministry of Science, ICT and the High value added Food Technology Development Program (Grant Number: 321051-5), funded by the Korea Institute of Planning & Evaluation for Technology in Food Agriculture, Food, & Fisheries, Republic of Korea.

## Data availability statement

Data included in article/supplementary material/referenced in article.

## Additional information

No additional information is available for this paper.

## CRediT authorship contribution statement

**Young-Bae Chung:** writing original draft; writing review & editing. **Sung Jin Park:** writing original draft; writing review & editing; Investigation; visualization. **Yun-Jeong Choi:** Formal analysis; Investigation; visualization. **Ye-Rang Yun:** Investigation; Methodology. **Mi-Ai Lee:** Data curation; Investigation; validation. **Sung Hee Park:** Conceptualization; Resources. **Sung Gi Min:** Project administration; Resources. **Hye-Young Seo:** Conceptualization; Resources.

## Declaration of competing interest

The authors declare the following financial interests/personal relationships which may be considered as potential competing interests:Hye-Young Seo reports financial support was provided by 10.13039/501100003722World Institute of Kimchi. Sung Gi Min reports financial support was provided by 10.13039/501100003722World Institute of Kimchi. If there are other authors, they declare that they have no known competing financial interests or personal relationships that could have appeared to influence the work reported in this paper.
